# Do influenza and pneumococcal vaccines prevent community-acquired respiratory infections among older people with diabetes and does this vary by chronic kidney disease? A cohort study using electronic health records

**DOI:** 10.1136/bmjdrc-2016-000332

**Published:** 2017-04-03

**Authors:** Helen I McDonald, Sara L Thomas, Elizabeth R C Millett, Jennifer Quint, Dorothea Nitsch

**Affiliations:** 1 Department of Non-Communicable Disease Epidemiology, London School of Hygiene and Tropical Medicine, London, UK; 2 Department of Infectious Disease Epidemiology, London School of Hygiene and Tropical Medicine, London, UK; 3 Department of Respiratory Epidemiology, Occupational Medicine and Public Health, National Heart and Lung Institute, Imperial College London, London, UK

**Keywords:** Adult Diabetes, Vaccine, Chronic Kidney Disease, Pneumococcal Infections

## Abstract

**Objective:**

We aimed to estimate the effectiveness of influenza and 23-valent pneumococcal polysaccharide vaccination on reducing the burden of community-acquired lower respiratory tract infection (LRTI) among older people with diabetes, and whether this varied by chronic kidney disease (CKD) status.

**Research design and methods:**

We used linked UK electronic health records for a retrospective cohort study of 190 492 patients ≥65 years with diabetes mellitus and no history of renal replacement therapy, 1997–2011. We included community-acquired LRTIs managed in primary or secondary care. Infection incidence rate ratios were estimated using the Poisson regression. Pneumococcal vaccine effectiveness (VE) was calculated as (1−effect measure). To estimate influenza VE, a ratio-of-ratios analysis (winter effectiveness/summer effectiveness) was used to address confounding by indication. Final VE estimates were stratified according to estimated glomerular filtration rate and proteinuria status.

**Results:**

Neither influenza nor pneumococcal vaccine uptake varied according to CKD status. Pneumococcal VE was 22% (95% CI 11% to 31%) against community-acquired pneumonia for the first year after vaccination, but was negligible after 5 years. In the ratio-of-ratios analysis, current influenza vaccination had 7% effectiveness for preventing community-acquired LRTI (95% CI 3 to 12). Pneumococcal VE was lower among patients with a history of proteinuria than among patients without proteinuria (p=0.04), but otherwise this study did not identify variation in pneumococcal or influenza VE by markers of CKD.

**Conclusions:**

The public health benefits of influenza vaccine may be modest among older people with diabetes. Pneumococcal vaccination protection against community-acquired pneumonia declines swiftly: alternative vaccination schedules should be investigated.

**Significance of this study**

**What is already known about this subject?**

- There is a large and growing burden of community-acquired lower respiratory tract infection and pneumonia among older people with diabetes, much of which can be directly or indirectly attributed to two vaccine-preventable pathogens: pneumococcus and influenza.

**What are the new findings?**

- We observed only a modest effectiveness of influenza vaccine against community-acquired lower respiratory tract infection (after adjustment for confounding by indication), while the effectiveness of pneumococcal vaccine against pneumonia waned over time. Our results suggested that pneumococcal vaccine effectiveness may be lower among patients with proteinuria but did not otherwise vary according to markers of chronic kidney disease.

**How might these results change the focus of research or clinical practice?**

- More effective immunization strategies and vaccination schedules may be needed for older people with diabetes.
- The low influenza vaccine effectiveness (VE) we observed against community-acquired lower respiratory tract infection, when contrasted with the large burden of infection directly and indirectly attributed to influenza, suggests scope for improved influenza immunization among this population, for example, with adjuvants.
- The suggestion of reduced pneumococcal VE among patients with proteinuria needs confirmation in a repeat study.

## Introduction

Hospital admissions for pneumonia are rising rapidly in the UK, most steeply among older people.^1^ Older people with diabetes have a particularly high burden of lower respiratory tract infection (LRTI), including pneumonia.^2^

Directly or indirectly, *Streptococcus pneumoniae* (‘pneumococcus’) and seasonal influenza viruses are responsible for a large burden of community-acquired pneumonia. The most common cause of community-acquired pneumonia is *S. pneumoniae*.^3^ Up to a third of community-acquired pneumonia may be influenza-related, due to bacterial coinfection or secondary bacterial pneumonia.^4^ Vaccination is available against both these pathogens, and recommended in the UK for everyone aged ≥65 years.^5^ However, the extent to which these vaccines protect against pneumonia among older people remains unclear for both vaccines.

The effectiveness of 23-valent pneumococcal polysaccharide vaccination against all-cause pneumonia among older people has been questioned, although meta-analyses have been hampered by between-study heterogeneity.^6^ ^7^ Waning immunity among vaccinated participants has been suggested as a possible cause, but few estimates are available of pneumococcal vaccine effectiveness (VE) according to time since vaccination.^8^

Traditional observational studies of influenza VE among older people may have overestimated influenza VE due to uncontrolled confounding by indication, in which the patient's functional status affects vaccine uptake.^8–12^ Observational studies which used strategies to control confounding by indication (such as a ‘ratio-of-ratios’ analysis in which the excess influenza VE during winter compared with summer is calculated) have suggested a null or modest influenza VE against community-acquired pneumonia among older people.^9^ ^13–15^

Older people with diabetes have a high prevalence of chronic kidney disease (CKD).^16^ Even at early stages, patients with CKD have increased incidence of LRTI and pneumonia.^16–18^ Patients with CKD have a generally reduced response to vaccines, and a faster decline in antibody levels following vaccination.^19^ Among patients receiving hemodialysis, a ratio-of-ratios analysis of influenza VE found no evidence of any protection against influenza-like-illness, influenza/pneumonia hospitalization, or mortality.^20^ Influenza VE at earlier stages of CKD is unclear, and still less is known about pneumococcal VE among patients with CKD.^19^ ^21^

We aimed to describe the extent to which the burden of community-acquired LRTI and pneumonia among older people with diabetes may be preventable with pneumococcal and influenza vaccination, and whether this varied according to CKD status. We conducted a retrospective cohort study using linked primary and secondary care electronic health record data to calculate the VE of pneumococcal vaccine against all community-acquired pneumonia. Since influenza vaccine may potentially reduce the incidence of influenza infection and secondary pneumonia, we calculated the influenza VE to prevent all community-acquired LRTI (considered as a broad category of all ‘chest infections’, including influenza infections, and possible secondary infections such as bronchitis and pneumonia), using a ratio-of-ratios analysis to address confounding by indication.

## Research design and methods

### Data sources

We analyzed data from the Clinical Practice Research Datalink (CPRD), a database of anonymized primary care medical records. Data were extracted in May 2011, and contained records for 12.8 million patients at 627 practices across the UK.^22^ Records include patient demographics, health behaviors, test results, diagnoses, and prescriptions. Diagnoses are recorded using Read codes, and have generally been found to have good positive predictive value in validations.^23^ The CPRD population is similar to the general UK population in terms of age and sex.^24^ ^25^

Linked data are available for patients in England, subject to practice-level consent. This study used linked data on all hospital inpatient admissions to NHS hospitals in England from Hospital Episodes Statistics (HES), and socioeconomic status from the Office for National Statistics (ONS).

### Study population

The study population comprised all patients in CPRD with diabetes mellitus, aged ≥65 years, with no history of renal replacement therapy, who had at least one valid serum creatinine result recorded in primary care. Diabetes was identified by diagnostic Read codes. For less definitive Read codes, we required confirmation with an antidiabetic medication prescription, as described in detail previously.^2^

Patients met eligibility criteria at the latest time-point of: diabetes diagnosis, 65th birthday, 1 year after practice registration, their general practice fulfilling CPRD quality control standards, or 1 April 1997. Their study entry date was their first valid serum creatinine result after the eligibility criteria were met. Patients left the study at the first time-point of: death, leaving the practice, last data collection from the practice, renal replacement therapy (dialysis or renal transplant), or 31 March 2011. Patients with a diagnosis of HIV or hyposplenia (including celiac disease or sickle cell disease) at any point in their medical record were excluded from the study.

### Definition of infections

LRTI was defined as a broad category of all infections of the lower respiratory tract, including influenza infections, bronchitis, and pneumonia.

A clinical diagnosis of infection was identified by a diagnostic Read code in primary care records, or a diagnostic International Classification of Disease 10 (ICD-10) code as the primary cause of hospital admission in secondary care records. To avoid overestimation from repeat attendances for the same infection, diagnostic codes recorded within 28 days of one another were attributed to a single episode of infection. The first consultation for infection was treated as the date of infection onset, and the infection had duration until 28 days after the latest of the last diagnostic code or hospital discharge. All infections with onset date during a HES hospitalization spell, or within 14 days following hospital discharge, or which included a code for postoperative infection, were designated hospital-acquired, and excluded. These methods have been described in detail previously.^26^

### Time at risk

Patients were not at risk of incident community-acquired infection during ongoing infection (community-acquired or hospital-acquired), during any hospitalization, or within 14 days following hospital discharge. These time periods were removed from time at risk. As pneumonia was a subset of LRTI, a patient could be at risk of pneumonia during an ongoing LRTI.

### Assignment of vaccination status

Vaccination status was identified from primary care records using Read codes, prescription data, and immunization record forms.

For pneumococcal vaccination, any of these records could define a first vaccination, and any subsequent prescription could identify a booster vaccination. Time-updated pneumococcal vaccination status was classified according to time since the latest pneumococcal vaccination (<1, 1–5, ≥5 years, never vaccinated).

Time-updated influenza vaccination status was assigned within vaccination years (1 September to 31 August). Within each vaccination year, influenza vaccination status was current from the first vaccination record to the subsequent 31 August. Patients without a current vaccination who had received an influenza vaccination within any of the previous five vaccination years were classified as having ‘residual’ influenza vaccination status, and other patients were categorized as unvaccinated.

### Definition of CKD

We studied two markers of CKD: estimated glomerular filtration rate (eGFR) and proteinuria. Estimated GFR was calculated from serum creatinine test results in primary care, using the CKD-EPI equation, including adjustment for black ethnicity.^27^ Estimated GFR status was time-updated using a last-carried-forward method, with eGFR status assigned according to the most recent creatinine result.^17^

A history of proteinuria was established from a Read code for persistent proteinuria or proteinuric disease, or a positive test result which did not coincide with a urinary tract infection diagnosis.

### Definitions for covariates

Age was categorized in 5-year bands up to a final category of ≥85 years. Socioeconomic status was assigned at a practice level, using 2007 ONS estimates of the Index of Multiple Deprivation, a composite area-level marker of deprivation.^28^ Smoking status was identified as current, ex-smoker, or non-smoker from HES or CPRD records. Comorbidities were identified from diagnostic Read codes in CPRD and were modeled as separate variables which were: ischemic heart disease, congestive cardiac failure, hypertension, cerebrovascular disease, other dementia, chronic lung disease (which included chronic obstructive pulmonary disease but not asthma), and chronic liver disease. Baseline HbA1C was defined by the most recent HbA1C test result in CPRD prior to (or on) the study entry date. Baseline medication history was identified from CPRD prescription records.

### Data analysis

Analysis was conducted separately for pneumococcal VE against pneumonia and for influenza VE against LRTI.

We excluded patients with missing smoking status. For comorbidities and proteinuria status, the absence of a positive record was treated as the absence of disease. The absence of a recorded HbA1C test result was included as indicating a relevant category of control.

Incidence rates and rate ratios were calculated for each infection using the Poisson regression with lexis expansions for age, and a random effects model to adjust for multiple infection episodes. We adjusted models for prespecified a priori confounders of the association between vaccination status and respiratory infection, and/or the relationship between CKD and respiratory infection. These were: age, sex, socioeconomic status at practice level, residential or nursing home care, baseline smoking status, time-updated comorbidities, steroid use in the 3 months prior to study entry, HbA1C and diabetic medication history at baseline, and date prior to or post 1 April 2004 (when Quality Outcomes Framework guidelines introduced financial incentives for recording CKD status among people with diabetes in primary care which may have improved ascertainment of CKD in primary care).^29^ No direct biological effect of ethnicity on VE was expected and so we did not adjust for this directly: instead, we adjusted for factors which may mediate any indirect confounding effect of ethnicity, such as CKD and other comorbidities and health behaviors.

For pneumococcal vaccine, VE was calculated as (1−effect estimate). To explore waning of immunity, we described pneumococcal VE according to time since vaccination.

To control for confounding by indication in influenza vaccination, we estimated the ratio of influenza VE in summer to influenza VE in winter in a ‘ratio-of-ratios’ analysis by including an interaction term between influenza vaccination status and season, and reporting the antilog of the β coefficient for the interaction term.^20^ Winter was defined as 1 September to 31 March, to capture excess winter influenza-like-illness.^30^

Final estimates of VE were stratified by time-updated eGFR and history of proteinuria, as markers of CKD.

Stata V.13.1 was used for data analyses. All code lists are available on request.

### Sensitivity analyses

Twenty-three-valent pneumococcal polysaccharide vaccination has been recommended for patients with CKD in the UK since 1992, but in 2003, the recommendation was extended to everyone aged ≥65 years.^5^ As a sensitivity analysis, we estimated pneumococcal VE separately for the periods before and after 31 March 2003 (to avoid separating the 2002–2003 winter season) to check for bias from secular changes in vaccine uptake.

The match of influenza vaccine strain to circulating influenza varies each year, which affects VE.^20^ As a sensitivity analysis, we estimated influenza VE separately for each winter. A further sensitivity analysis defined the start of the influenza season as the first week after 1 September in which weekly influenza-like illness incidence in primary care exceeded 30/100 000 people, limited to the years 2004–2011 due to data availability.^31^

We also conducted a sensitivity analysis of influenza VE excluding patients with chronic lung disease or congestive heart failure, as the relationship of influenza to LRTI etiology for these patients may differ from that among the general population.

For both vaccines, we conducted a sensitivity analysis limited to patients with white ethnicity.

### Ethics

The study was approved by the Independent Scientific Advisory Group of the CPRD (ISAC reference 11_033A) and the London School of Hygiene and Tropical Medicine Ethics Committee (LSHTM reference 6116).

## Results

**Figure 1:**
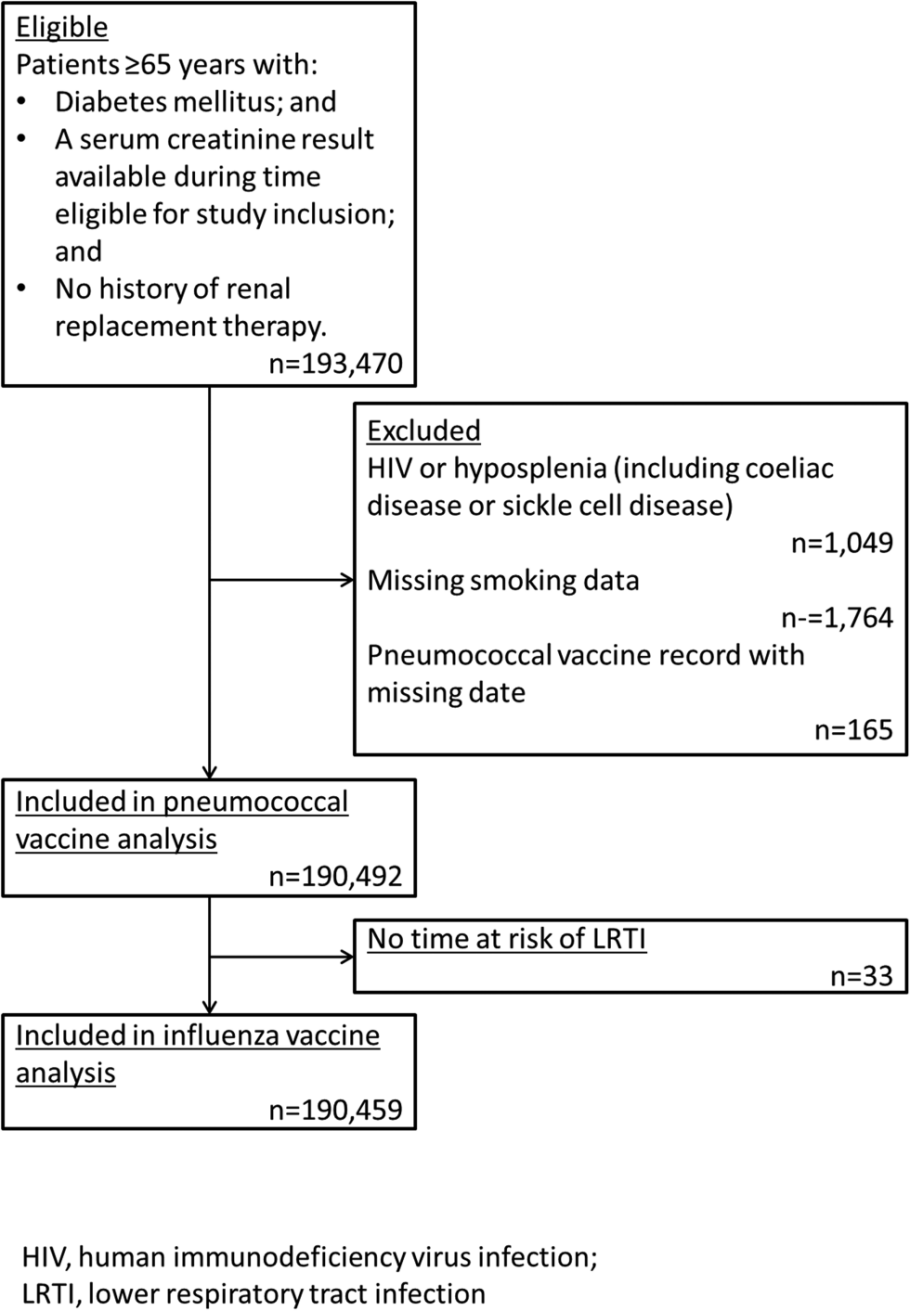
Flow chart of study inclusion. LRTI, lower respiratory tract infection.

Of 193 470 eligible patients, 1049 patients (0.5%) with a diagnosis of HIV or hyposplenia, 1764 (0.9%) patients with no smoking status available, and 165 (<0.1%) patients who had a record of pneumococcal vaccine administration with a missing date were excluded from both analyses (figure 1). For pneumococcal and influenza vaccinations, unvaccinated patients had a lower recorded prevalence of ischemic heart disease and chronic lung disease than vaccinated patients. Unvaccinated patients may have had poorer diabetic control than vaccinated patients: a higher proportion had poor or unrecorded HbA1C status, and a lower proportion had a history of oral antidiabetic medication and insulin prescription than vaccinated patients. The prevalence of CKD was similar for vaccinated and unvaccinated patients, although unvaccinated patients had a slightly lower prevalence of a recorded history of proteinuria (table 1).

**Table 1:** Baseline description of study population

		Pneumococcal vaccine status at baseline n=190 492		Influenza vaccine status at baseline n=190 459		
		Never vaccinated n=79 476	Vaccinated n=111 016	Unvaccinated* n=32 552	Currently vaccinated n=124 130	Residual 1–5 years n=33 777
Age (years)		71 (66–77)	72 (66–78)	71 (66–77)	72 (66–78)	71 (66–78)
Median (IQR)		n (%)	n (%)	n (%)	n (%)	n (%)
Female		40 308 (50.7)	53 146 (47.9)	16 603 (51.0)	60 019 (48.4)	16 813 (49.8)
Socioeconomic status†						
1 (least deprived)		13 701 (17.2)	19 912 (17.9)	5618 (17.3)	22 181 (17.9)	5809 (17.2)
2		14 666 (18.5)	19 591 (17.7)	5799 (17.8)	22 394 (18.0)	6058 (17.9)
3		16 156 (20.3)	23 329 (21.0)	6567 (20.2)	25 957 (20.9)	6956 (20.6)
4		17 758 (22.3)	25 481 (23.0)	7312 (22.5)	28 128 (22.7)	7789 (23.1)
5 (most deprived)		17 195 (21.6)	22 703 (20.5)	7256 (22.3)	25 470 (20.5)	7165 (21.2)
Ethnicity						
White		43 357 (54.6)	63 577 (57.3)	17 037 (52.3)	71 268 (57.4)	18 615 (55.1)
South Asian		1515 (1.9)	2353 (2.1)	429 (1.3)	2606 (2.1)	833 (2.5)
Black		923 (1.2)	1167 (1.1)	343 (1.1)	1290 (1.0)	457 (1.4)
Other		636 (0.8)	717 (0.7)	225 (0.7)	860 (0.7)	267 (0.8)
Missing		33 045 (41.6)	43 202 (38.9)	14 518 (44.6)	48 106 (38.8)	13 605 (40.3)
Residential care		1697 (2.1)	3274 (3.0)	436 (1.3)	3516 (2.8)	1016 (3.0)
Smoking status						
Non-smoker		38 078 (47.9)	44 713 (40.3)	15 449 (47.5)	52 782 (42.5)	14 543 (43.1)
Current smoker		13 901 (17.5)	16 439 (14.8)	6252 (19.2)	18 327 (14.8)	5756 (17.0)
Ex-smoker		27 497 (34.6)	49 864 (44.9)	10 851 (33.3)	53 021 (42.7)	13 478 (39.9)
Comorbidities						
Ischemic heart disease		18 886 (23.8)	34 415 (31.0)	6825 (21.0)	36 761 (29.6)	9713 (28.8)
Congestive cardiac failure		5935 (7.5)	10 018 (9.0)	2175 (6.7)	10 721 (8.6)	3065 (9.1)
Hypertension		46 626 (58.7)	71 311 (64.2)	18 644 (57.3)	78 252 (63.0)	21 024 (62.2)
Cerebrovascular disease		9714 (12.2)	14 469 (13.0)	3612 (11.1)	16 021 (12.9)	4540 (13.4)
Other dementia		1437 (1.8)	1956 (1.8)	322 (1.0)	2326 (1.9)	729 (2.2)
Chronic lung disease		4016 (5.1)	10 881 (9.8)	1515 (4.7)	10 530 (8.5)	2851 (8.4)
Chronic liver disease		402 (0.5)	734 (0.7)	168 (0.5)	729 (0.6)	240 (0.7)
Steroid use in previous 3 months		2870 (3.6)	5560 (5.0)	1039 (3.2)	5841 (4.7)	1544 (4.6)
Latest HbA1C status % (mmol/mol)						
None recorded		11 202 (14.1)	10 620 (9.6)	4872 (15.0)	13 317 (10.7)	3627 (10.7)
Good <7% (<53)		34 669 (43.6)	53 305 (48.0)	13 621 (41.8)	58 741 (47.3)	15 596 (46.2)
Intermediate 7–10% (53–86)		27 935 (35.2)	41 354 (37.3)	11 389 (35.0)	45 383 (36.6)	12 509 (37.0)
Poor >10% (>86)		5670 (7.1)	5737 (5.2)	2670 (8.2)	6689 (5.4)	2045 (6.1)
Antidiabetes medication history						
None		38 755 (48.8)	50 517 (45.5)	16 463 (50.6)	58 195 (46.9)	14 598 (43.2)
Oral		33 623 (42.3)	46 949 (42.3)	13 613 (41.8)	51 914 (41.8)	15 031 (44.5)
Insulin		2889 (3.6)	4136 (3.7)	1024 (3.2)	4687 (3.8)	1314 (3.9)
Oral and insulin		4209 (5.3)	9414 (8.5)	1452 (4.5)	9334 (7.5)	2834 (8.4)
Latest eGFR mL/min/1.73 m^2^						
<30		2098 (2.6)	2986 (2.7)	767 (2.4)	3337 (2.7)	977 (2.9)
30–44		7558 (9.5)	10 607 (9.6)	2932 (9.0)	11 964 (9.6)	3254 (9.6)
45–59		18 678 (23.5)	25 508 (23.0)	7337 (22.5)	29 039 (23.4)	7806 (23.1)
≥60		51 142 (64.4)	71 915 (64.8)	21 516 (66.1)	79 790 (64.3)	21 740 (64.4)
History of proteinuria						
No		71 095 (89.5)	94 128 (84.8)	29 231 (89.8)	107 212 (86.4)	28 735 (85.1)
Yes		8381 (10.6)	16 888 (15.2)	3321 (10.2)	16 918 (13.6)	5042 (14.9)

*Not vaccinated within the five previous years.

†Index of multiple deprivation quintile for primary care practice.

eGFR, estimated glomerular filtration rate.

### Pneumococcal vaccine

A total of 190 492 patients contributed 811 498 person-years to the pneumococcal vaccine analysis, during which there were 7805 community-acquired pneumonia episodes among 7036 people. At study entry, 58.3% of patients (111 016/190 492) were vaccinated against pneumococcal disease (table 1). Baseline pneumococcal vaccination increased among patients who entered the study after 2003–2004, and did not differ according to eGFR at baseline (see online supplementary figure S1A).

10.1136/bmjdrc-2016-000332.supp1 supplementary data

Crude rates of pneumonia were lowest among patients within a year of pneumococcal vaccine. The adjusted effectiveness of pneumococcal vaccine for preventing pneumonia was 22% (95% CI 11% to 31%) within the first year after vaccination, and fell with increasing time since vaccination. Pneumonia incidence among patients vaccinated more than 5 years previously was similar to that among patients with no record of vaccination (incidence rate ratio, IRR 1.03: 95% CI 0.95 to 1.11). There was the suggestion of a trend of decreased pneumococcal VE among patients with reduced eGFR, but this was not statistically significant. There was a greater protective effect of pneumococcal vaccine among patients without a history of proteinuria than with a history of proteinuria (table 2).

**Table 2:** Pneumococcal vaccine effectiveness against pneumonia (n=190 492)

	Pneumococcal vaccination status			
	Never	<1 year	1–4 years	≥5 years
Person-time (years)	189 776	51 397	275 841	294 484
Infections (n)	1661	326	2255	3563
Crude pneumonia rate/1000 person-years (95% CI)	9.0 (8.6 to 9.5)	6.6 (5.9 to 7.3)	8.7 (8.3 to 9.1)	13.6 (13.1 to 14.1)
Adjusted* pneumonia rate ratio (95% CI)	1 (reference)	0.78 (0.69 to 0.89)	0.92 (0.85 to 0.99)	1.03 (0.95 to 1.11)
Vaccine effectiveness* % (95% CI)	0 (reference)	22 (11 to 31)	8 (1 to 15)	−3 (−11 to 5)
Vaccine effectiveness* % (95% CI) stratified by eGFR status (mL/min/1.73 m^2^)				
eGFR <30	0 (reference)	6 (−40 to 37)	4 (−22 to 25)	6 (−19 to 26)
eGFR 30–44	0 (reference)	16 (−12 to 37)	1 (−18 to 17)	−7 (−27 to 11)
eGFR 45–59	0 (reference)	21 (−1 to 38)	9 (−6 to 21)	−1 (−17 to 14)
eGFR ≥60	0 (reference)	26 (11 to 39)	12 (2 to 22)	−3 (−15 to 8)
p Value (test for trend)†	–	0.25	0.49	0.07
Vaccine effectiveness* % (95% CI) stratified by proteinuria status				
No proteinuria	0 (reference)	28 (16 to 38)	13 (5 to 20)	1 (−8 to 10)
Proteinuria	0 (reference)	2 (−25 to 23)	−6 (−23 to 9)	−19 (−38 to −3)
p Value (interaction)‡	–	0.04	0.03	0.04

*Adjusted for: age, sex, socioeconomic status at practice level, residential care, date post 1 April 2004, smoking status, time-updated comorbidities (ischemic heart disease, congestive cardiac failure, hypertension, cerebrovascular disease, other dementia, chronic lung disease, chronic liver disease), time-updated CKD status (eGFR, proteinuria), steroid use in the 3 months prior to study entry, influenza vaccination status, and HbA1C and diabetic medication history at baseline.

†Wald test for interaction term of pneumococcal vaccine with eGFR.

‡Wald test for interaction term of pneumococcal vaccine with proteinuria.

A sensitivity analysis of pneumococcal VE stratified by date before or after 1 April 2003 suggested that the estimate was not affected by the change in vaccine recommendation in 2003 (see online supplementary table S1).

### Influenza vaccine

For the influenza VE analysis, 190 459 patients contributed 803 230 person-years to time at risk, during which there were 114 313 cases of LRTI among 55 685 patients. At study entry, 65.2% of patients (124 130/190 459) had received a current vaccination against influenza (table 1). Baseline influenza vaccination status increased slightly over time, and did not differ by eGFR status (see online supplementary figure S1B).

Vaccinated patients had a higher crude incidence of LRTI than unvaccinated patients, in winter and summer. After adjustment for age, sex, comorbidities, pneumococcal vaccination, and characteristics of diabetes, the winter incidence rate of LRTI was higher among patients with a current influenza vaccine than unvaccinated patients (IRR 1.19: 95% CI 1.15 to 1.23) and among patients with residual influenza vaccination than unvaccinated patients (IRR 1.23: 95% CI 1.18 to 1.28). Similar or higher, adjusted IRRs were observed in summer. Using the ratio-of-ratios analysis, a 7% effectiveness of current influenza vaccine (95% CI 3 to 12) and a 12% effectiveness of residual influenza vaccination (95% CI 7 to 17) to prevent community-acquired LRTI were observed. There was no evidence to suggest a relationship between VE and eGFR nor proteinuria (table 3).

**Table 3:** Lower respiratory tract infection (LRTI) rates and influenza vaccine effectiveness to prevent LRTI by season (n=190 459)

	Summer			Winter		
	Influenza vaccination status			Influenza vaccination status		
	>5 years/never	Current	Residual 1–5 years	>5 years/never	Current	Residual 1–5 years
Person-time (years)	35 233	219 456	74 704	47 352	355 766	70 718
Infections (n)	2363	22 726	8496	5751	62 077	12 900
Crude LRTI rate /1000 py (95% CI)	73.0 (69.5 to 76.5)	111.0 (109.2 to 112.9)	121.3 (118.4 to 124.1)	134.8 (130.3 to 139.2)	187.2 (185.4 to 189.4)	195.5 (191.6 to 199.4)
Crude LRTI rate ratio (95% CI)	1 (ref)	1.52 (1.45 to 1.60)	1.66 (1.58 to 1.75)	1 (ref)	1.38 (1.34 to 1.44)	1.45 (1.40 to 1.51)
Adjusted* LRTI rate ratio (95% CI)						
Overall	1 (ref)	1.28 (1.21 to 1.35)	1.39 (1.32 to 1.47)	1 (ref)	1.19 (1.15 to 1.23)	1.23 (1.18 to 1.28)
Stratified by eGFR (mL/min/1.73 m^2^)						
eGFR <30	1 (ref)	1.20 (0.94 to 1.52)	1.29 (1.01 to 1.65)	1 (ref)	1.17 (0.99 to 1.38)	1.20 (1.01 to 1.43)
eGFR 30–44	1 (ref)	1.27 (1.10 to 1.45)	1.31 (1.13 to 1.51)	1 (ref)	1.20 (1.10 to 1.32)	1.20 (1.08 to 1.33)
eGFR 45–59	1 (ref)	1.32 (1.19 to 1.46)	1.40 (1.26 to 1.56)	1 (ref)	1.16 (1.09 to 1.25)	1.20 (1.12 to 1.30)
eGFR ≥ 60	1 (ref)	1.27 (1.19 to 1.37)	1.42 (1.32 to 1.53)	1 (ref)	1.21 (1.15 to 1.27)	1.26 (1.20 to 1.33)
Stratified by proteinuria						
No proteinuria	1 (ref)	1.27 (1.20 to 1.35)	1.36 (1.30 to 1.45)	1 (ref)	1.18 (1.13 to 1.23)	1.22 (1.16 to 1.27)
Proteinuria	1 (ref)	1.33 (1.19 to 1.49)	1.50 (1.33 to 1.68)	1 (ref)	1.23 (1.14 to 1.33)	1.27 (1.17 to 1.38)
Ratio of incidence rate ratios* winter/summer (95% CI)						
Overall				1 (ref)	0.93 (0.88 to 0.97)	0.88 (0.83 to 0.93)
Stratified by eGFR (mL/min/1.73 m^2^)						
eGFR <30				1 (ref)	0.93 (0.73 to 1.17)	0.90 (0.70 to 1.16)
eGFR 30–44				1 (ref)	0.88 (0.77 to 1.01)	0.86 (0.75 to 1.00)
eGFR 45–59				1 (ref)	0.90 (0.81 to 0.99)	0.87 (0.78 to 0.98)
eGFR ≥ 60				1 (ref)	0.95 (0.89 to 1.02)	0.89 (0.83 to 0.96)
Stratified by proteinuria						
No proteinuria				1 (ref)	0.93 (0.88 to 0.99)	0.90 (0.84 to 0.95)
Proteinuria				1 (ref)	0.90 (0.81 to 1.00)	0.83 (0.74 to 0.93)
Vaccine effectiveness (VE)* based on ratio of incidence rate ratios % (95% CI)						
Overall				0 (ref)	7 (3 to 12)	12 (7 to 17)
Stratified by eGFR (mL/min/1.73 m^2^)						
eGFR <30				0 (ref)	7 (−17 to 27)	10 (−16 to 30)
eGFR 30–44				0 (ref)	12 (−1 to 23)	14 (0 to 25)
eGFR 45–59				0 (ref)	10 (1 to 19)	13 (2 to 22)
eGFR ≥ 60				0 (ref)	5 (−2 to 11)	11 (4 to 17)
p Value (test for trend)†				–	0.31	0.79
Stratified by proteinuria						
No proteinuria				0 (ref)	7 (1 to 12)	10 (5 to 16)
Proteinuria				0 (ref)	10 (0 to 19)	17 (7 to 26)
p Value‡				–	0.56	0.26

*Adjusted for: age, sex, socioeconomic status at practice level, residential care, date post 1 April 2004, smoking status, time-updated comorbidities (ischemic heart disease, congestive cardiac failure, hypertension, cerebrovascular disease, other dementia, chronic lung disease, chronic liver disease), time-updated CKD status (eGFR, proteinuria), steroid use in the 3 months prior to study entry, pneumococcal vaccination, and HbA1C and diabetic medication history at baseline.

†Wald test for interaction of eGFR with influenza vaccination status and season, with eGFR modeled as a linear variable.

‡Wald test for interaction of proteinuria with influenza vaccine and season.

LRTI, lower respiratory tract infection; py, person-years.

Similar results were obtained in sensitivity analyses of influenza VE stratified by year (see online supplementary table S2), and excluding patients with chronic lung disease and congestive heart failure (see online supplementary table S3). Analyses using the influenza season dates did not change the results materially and are not shown.

Sensitivity analysis limited to patients with white ethnicity did not change the results for either vaccine (results not shown).

## Conclusions

Influenza and pneumococcal vaccine uptake was high among this study population of older people with diabetes mellitus, and did not vary according to markers of CKD. Pneumococcal vaccine had 22% (95% CI 11% to 31%) effectiveness against community-acquired pneumonia within the first year after vaccination. Pneumonia incidence among patients vaccinated more than 5 years previously was similar to that among patients with no record of vaccination (IRR 1.03: 95% CI 0.95 to 1.11). Community-acquired LRTI rates were higher among patients who received an influenza vaccination than among patients who did not, and this relationship remained after adjustment for age, sex, comorbidities, and characteristics of diabetes, and was observed in summer and winter. Traditional analyses would have concluded that influenza vaccination is associated with community-acquired LRTI. However, using the ratio-of-ratios analysis, a 7% effectiveness (95% CI 3% to 12%) of current influenza vaccine against community-acquired LRTI was observed. There was no evidence of a trend in influenza VE according to CKD status. However, there was evidence for a greater protective effect of pneumococcal vaccine among patients without a history of proteinuria than patients with a history of proteinuria.

Previous meta-analyses have found insufficient evidence for a protective effect of pneumococcal vaccine against all-cause pneumonia among the adult population due to heterogeneity.^6^ ^7^ A subgroup analysis of a large Spanish cohort study found that only recent pneumococcal vaccination (<5 years) protected against hospitalization for all-cause community-acquired pneumonia (HR 0.75; 95% CI 0.58 to 0.98) among the general population aged ≥60 years.^8^ The authors suggested that the heterogeneity observed in meta-analyses might be explained by waning immunity among the vaccinated population. Our results support this view and suggest that pneumococcal vaccination appears to be effective against all-cause community-acquired pneumonia for a year following vaccination among people aged ≥65 years with diabetes, after which time we observed a decrease in pneumococcal VE to a null effect after 5 years.

Previous cohort studies among older people have provided evidence of a ‘healthy vaccinee effect’, in which higher vaccine uptake among healthier patients resulted in likely overestimation of influenza VE.^9^ ^10^ ^12^ ^32^ ^33^ Evidence suggesting a healthy vaccinee effect has also previously been found among older people with diabetes.^34–38^ In contrast, we observed higher rates of LRTI among patients who had received an influenza vaccination than among unvaccinated patients: our vaccinated patients appear, on this outcome measure, to be *less* healthy than unvaccinated patients. This finding is intriguing. The major difference between our study and most previous studies of this question is that we have included community-acquired LRTIs diagnosed and managed in primary and secondary care. One possible explanation of the difference is that vaccination may reflect health-seeking behavior in primary care. When patients develop symptoms of LRTI, patients who attend primary care for diagnosis and treatment may also be patients who were more likely to take up the influenza vaccine. This ascertainment bias may be less relevant to studies with hospitalization as an outcome—or could even be reversed, as vaccinated patients who attended primary care promptly with LRTI may be less likely to require hospital admission. An alternative explanation is that the healthy vaccinee effect observed in studies of hospitalization for LRTI/pneumonia may reflect residual confounding by ‘frailty’ in which frailer patients are less likely to take up vaccination and more likely to be admitted to hospital when they develop infection. This would be less relevant to diagnosis of LRTI in primary care, and so our outcome may be less vulnerable to residual confounding by indication.

Our ‘ratio-of-ratios’ estimate suggested 7% VE of current influenza vaccination against LRTI among older people with diabetes (95% CI 3 to 12). Previous studies using similar strategies among the general population of older people have found no evidence of influenza VE against community-acquired pneumonia (VE 8%: 95% CI −10% to 23%), and evidence of a modest protection against influenza-related excess hospitalization with pneumonia/influenza (VE 19%: 95% CI 4% to 31%).^14^ ^15^ Our estimate is consistent with both these estimates, and the difference may be due to the higher precision available for the present study due to the large cohort size.

Our results suggested that pneumococcal VE may be reduced among patients with a history of proteinuria. We did not find any evidence of altered influenza VE among patients with CKD, but this may be due to limited power for the stratified ratio-of-ratios analysis. To the best of our knowledge, neither pneumococcal VE against pneumonia nor influenza VE against LRTI using methods to control for confounding by indication has been studied among patients with CKD who are not receiving dialysis. Studies of patients receiving dialysis may give some indication as to whether alteration of VE with CKD status is likely. A large observational study of pneumococcal vaccine found no evidence of effectiveness against hospitalization for pneumonia or respiratory infections among patients receiving dialysis.^39^ A study of influenza vaccine which calculated a ratio-of-ratios VE comparing influenza effectiveness in years with good match between the vaccine and circulating strain to effectiveness in a poorly matched ‘placebo year’ found no evidence of protection against influenza/pneumonia hospitalization among patients receiving hemodialysis (VE 2%: 95% CI −2% to5%).^20^ These studies suggest that the suggestion of reduced pneumococcal VE associated with CKD is plausible, but this question requires further investigation before conclusions can be drawn.

This study has several strengths. We used large, linked data sets with a careful definition of infection episodes to identify community-acquired infections managed in primary or secondary care, and excluded hospital-acquired infections and hospitalization from time at risk. This avoids differential hospital attendance patterns biasing estimates of VE according to markers of CKD. We adjusted for a wide range of comorbidities, and conducted a ratio-of-ratios analysis for influenza VE to address confounding by indication. We described the effect of pneumococcal vaccine according to time since vaccination, including booster doses, to identify waning immunity following vaccination. Our study population of older people with diabetes is well monitored for CKD,^40^ and this permitted us to explore the relationship of influenza and pneumococcal VE with CKD among patients not receiving dialysis, which we believe is novel.

As an observational study of VE using routinely collected health record data, the study has limitations*.* LRTI/pneumonia is typically diagnosed clinically in general practice, without microbiological testing for the causative pathogen. Thus, we chose broader LRTI/pneumonia outcomes, in common with previous observational studies of influenza VE. We may have underascertained proteinuria and comorbidities; however, the selection of a highly monitored study population should minimize this risk, and the high prevalence of each we observed suggests that this was not a major source of misclassification. Despite adjustment for multiple comorbidities, residual confounding by indication may remain in the pneumococcal VE analysis. Despite our use of large, linked data sets, we had limited power to estimate the relationship of VE according to CKD status, especially in a ratio-of-ratios influenza VE analysis.

Our findings have implications for clinical practice, public health, and future research. Our results should not be interpreted as demonstrating that influenza vaccine is ineffective among this population. We did not study the effectiveness of either vaccine against infection with their specific pathogens. As such, the results should neither discourage patients nor health professionals from influenza and pneumococcal vaccination.

Our study question was the extent to which the burden of community-acquired LRTI may be preventable with vaccination and our results suggest that the growing burden of community-acquired LRTI and pneumonia among this population cannot be easily tackled by increasing uptake of existing routine vaccination programs. This is relevant for public health—in planning health service provision and designing effective strategies to prevent illness. It should also prompt a call for research into more effective immunization strategies and vaccination schedules. The low influenza VE we observed against community-acquired LRTI, when contrasted with the large burden of infection directly and indirectly attributed to influenza, suggests scope for strategies to improve vaccination effectiveness and better immunization among this population, for example, the use of adjuvants in vaccines. The suggestion of reduced pneumococcal VE among patients with proteinuria is interesting and needs confirmation in a repeat study.
