# Supplementary material for: Do influenza and pneumococcal vaccines prevent community-acquired respiratory infections among older people with diabetes and does this vary by chronic kidney disease? A cohort study using electronic health records
Source: BMJ Open Diabetes Res Care. 2017 Apr 3;5(1):e000332. doi: 10.1136/bmjdrc-2016-000332 (PMC5387965; doi:10.1136/bmjdrc-2016-000332)

Supplemental Table S1: Association of pneumococcal vaccine status with community-acquired pneumonia incidence before and after 1 April 2003 (n=190,492)

Adjusted [1] pneumonia rate ratio (95% CI)	Pneumococcal vaccination status			
	Never	< 1 year	1–4 years	≥ 5 years
1 April 1997 – 31 March 2003	1 (ref)	0.76 (0.59–0.99)	0.88 (0.75–1.02)	0.97 (0.79–1.18)
1 April 2003 – 31 March 2011	1 (ref)	0.78 (0.68–0.90)	0.92 (0.84–1.01)	1.03 (0.94–1.12)

1. Adjusted for: age, sex, socio-economic status at practice level, residential care, smoking status, time-updated co-morbidities (ischaemic heart disease, congestive cardiac failure, hypertension, cerebrovascular disease, other dementia, chronic lung disease, chronic liver disease), time-updated CKD status (eGFR, proteinuria), steroid use in the 3 months prior to study entry, influenza vaccination status, and HbA1C and diabetic medication history at baseline.

Supplemental Table S2: association of influenza vaccine status with community-acquired LRTI incidence for each winter (n=190,459)

Year	Number of LRTIs in winter	Adjusted [1] LRTI incidence rate ratio (95% CI) according to influenza vaccination status in winter [2]		
		>5 years/ never	Current	Residual 1-5 years
1997-8	620	1 (ref)	1.33 (1.03–1.72)	1.45 (1.08–1.94)
1998-9	1,137	1 (ref)	1.45 (1.22–1.77)	1.37 (1.11–1.70)
1999-2000	1,821	1 (ref)	1.23 (1.06–1.42)	1.24 (1.05–1.47)
2000-1	2,583	1 (ref)	1.21 (1.05–1.39)	1.40 (1.17–1.66)
2001-2	3,449	1 (ref)	1.33 (1.16–1.53)	1.44 (1.22–1.69)
2002-3	4,641	1 (ref)	1.18 (1.04–1.34)	1.30 (1.13–1.50)
2003-4	6,266	1 (ref)	1.35 (1.20–1.51)	1.52 (1.33–1.73)
2004-5	7,656	1 (ref)	1.23 (1.11–1.37)	1.19 (1.05–1.34)
2005-6	8,084	1 (ref)	1.14 (1.01–1.28)	1.14 (1.00–1.28)
2006-7	8,666	1 (ref)	1.17 (1.05–1.32)	1.09 (0.97–1.23)
2007-8	9,134	1 (ref)	1.12 (0.99–1.25)	1.20 (1.07–1.35)
2008-9	10,061	1 (ref)	1.24 (1.11–1.38)	1.25 (1.11–1.40)
2009-10	8,945	1 (ref)	1.33 (1.18–1.49)	1.42 (1.25–1.61)
2010-11	7,661	1 (ref)	1.12 (0.99–1.26)	1.16 (1.02–1.31)

1. Adjusted for: age, sex, socio-economic status at practice level, residential care, smoking status, time-updated co-morbidities (ischaemic heart disease, congestive cardiac failure, hypertension, cerebrovascular disease, other dementia, chronic lung disease, chronic liver disease), time-updated CKD status (eGFR, proteinuria), steroid use in the 3 months prior to study entry, pneumococcal vaccination, and HbA1C and diabetic medication history at baseline.
2. Winter was defined as 1 September to 31 March.

Supplemental Table S3: Adjusted LRTI rate ratio according to influenza vaccination status excluding patients with congestive heart failure or chronic lung disease (n=162,412)

	Summer			Winter		
	Influenza vaccination status			Influenza vaccination status		
	>5 years/ never	Current	Residual	>5 years/ never	Current	Residual
Person-time (years)	30,904	182,214	61,928	41,582	295,921	58,684
Infections	1,578	13,587	5,020	3,976	39,304	8,054
Adjusted[1] LRTI rate ratio (95% CI)	1 (ref)	1.32 (1.24–1.41)	1.42 (1.32–1.51)	1 (ref)	1.23 (1.17–1.28)	1.25 (1.19–1.31)
Ratio of incidence rate ratios[1] winter/summer (95% CI)				1 (ref)	0.95 (0.89–1.01)	0.90 (0.84–0.96)
VE[1] based on ratio of incidence rate ratios % (95% CI)				0 (ref)	5 (-1–11)	10 (4–16)

LRTI, lower respiratory tract infection; VE, vaccine effectiveness

2. Adjusted for: age, sex, socio-economic status at practice level, residential care, date post 1 April 2004, smoking status, time-updated co-morbidities (ischaemic heart disease, hypertension, cerebrovascular disease, other dementia, chronic liver disease), time-updated CKD status (eGFR, proteinuria), steroid use in the 3 months prior to study entry, pneumococcal vaccination, and HbA1C and diabetic medication history at baseline.

Supplemental Figure S1: Baseline vaccination status over time

S1A: Pneumococcal vaccination status at baseline according to year of study entry and estimated glomerular filtration rate status (eGFR)

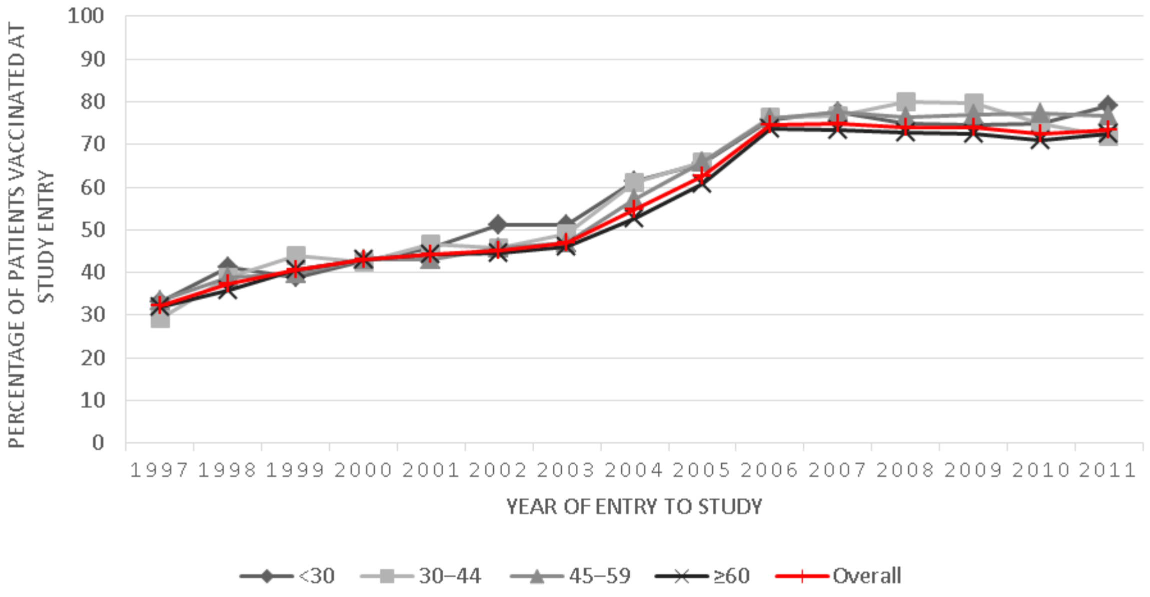

S1B: Influenza vaccination status at baseline according to year of study entry and estimated glomerular filtration rate status (eGFR)

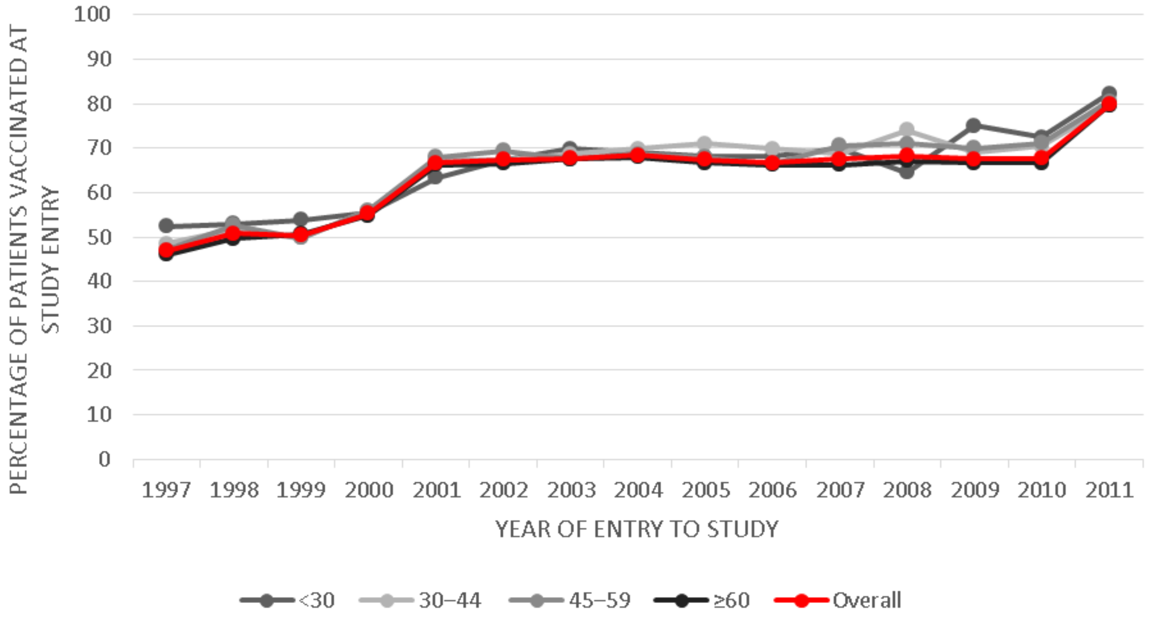

Supplement: supplementary data [file bmjdrc-2016-000332supp.pdf]
